# 1184. Investigating the Relationship Between Beta-Lactam Antibiotics and Thrombocytopenia: A Propensity Score-Matched Cohort Study

**DOI:** 10.1093/ofid/ofad500.1024

**Published:** 2023-11-27

**Authors:** Alyssa Y Chen, John J Hanna, Mujeeb Basit, Marguerite Monogue

**Affiliations:** Mount Sinai Hospital, Dallas, Texas; University of Texas Southwestern, Dallas, Texas; UT Southwestern Medical Center, Dallas, Texas; University of Texas Southwestern Medical Center, Dallas, TX

## Abstract

**Background:**

Cases of beta-lactam (BL) induced thrombocytopenia (TCP) have been reported, but the evidence on whether BLs pose an increased risk for TCP remains inconclusive. This large-scale study examines the association of TCP with BLs when compared to alternative non-beta-lactams (nBL).

**Methods:**

This retrospective study included adult inpatients who received at least one antibiotic administration at The University of Texas Southwestern Medical Center between 2008 and 2021. We excluded surgical patients, patients with baseline TCP, noncontinuous antibiotic therapy, no recorded platelet count value following antibiotic administration, and those who received more than one class of antibiotic therapy. Antibiotics were grouped into classes and subclasses (Table 1). BL as well as each BL subclass were compared to nBL. For each comparison group, propensity score matching was performed to control for age, sex, baseline PLT, days of antibiotic therapy, immunosuppression, exposure to other thrombocytopenic medications, hematologic cancer, and transplant status. The primary outcome was TCP defined as 1)< 150×10^9^/L or 2) > 50% decrease from baseline in the 30 days following the first antibiotic administration. Secondary outcomes included TCP in 3 and 7 days, and clinically significant TCP(< 50×109/L).

**Figure:**
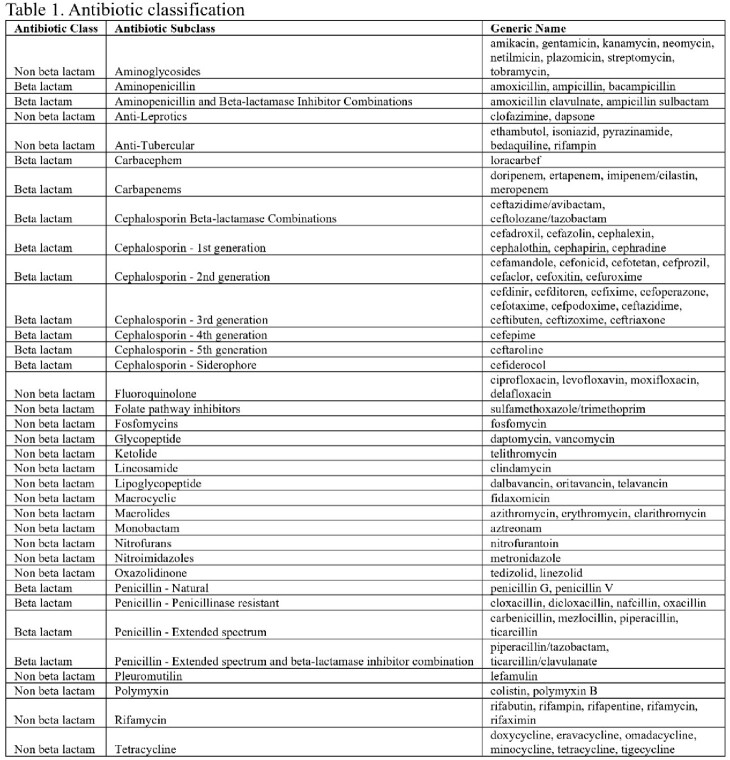

**Results:**

A total of 13,887 unique patients were included, with 6,127 received exclusively BL and 7,760 nBL (Figure 1). Covariate balance was achieved following matching for each comparison. After matching, 931/5047 patients in the BL group developed TCP compared to 875/ 5047 matched patients in the nBL group. There was no statistically significant difference in the odds of TCP between both groups (OR, 1.08; 95 CI%, 0.97-1.19). Of the BL subclasses, only 1st generation cephalosporins and extended combination penicillins were found to carry statistically significant greater odds of TCP compared to nBL (Table 2). However, these differences disappeared for clinically significant TCP levels. There was no statistically significant difference in time to TCP for BL compared to nBL (Figure 2).

**Figure 1.**
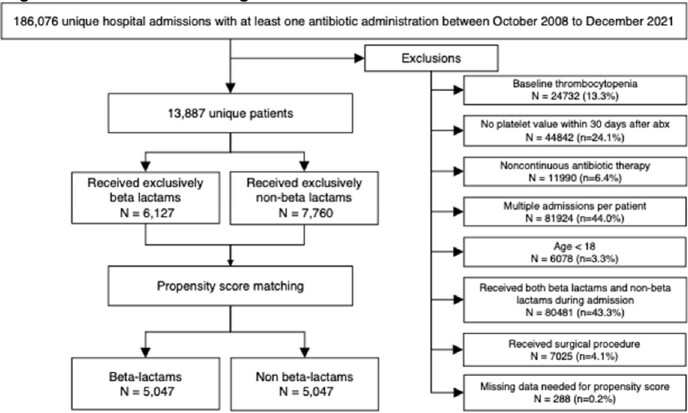
Cohort flow diagram

**Figure:**
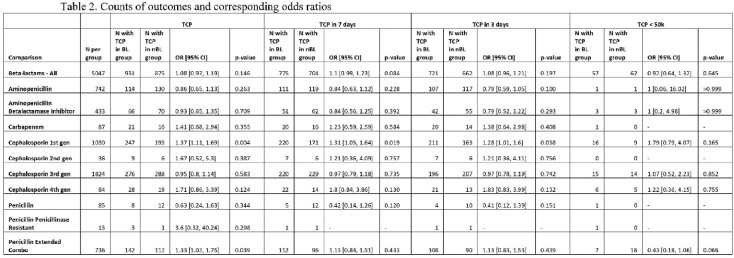

**Figure 2.**
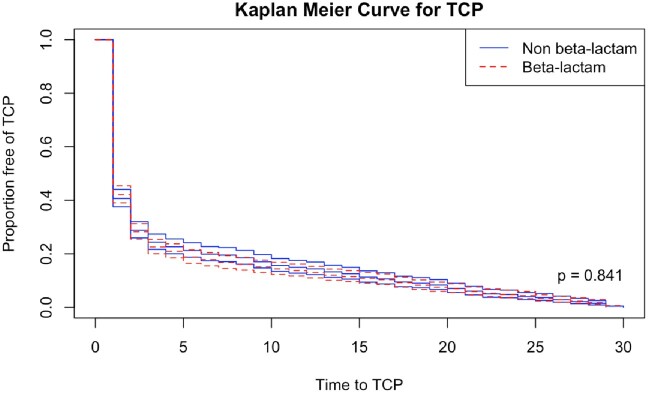
Kaplan Meier curve for thrombocytopenia by antibiotic class

**Conclusion:**

The study found no statistically significant increase in TCP risk for BL when compared to nBL. Therefore, the risks and benefits should be considered before switching off preferred BL therapy.

**Disclosures:**

**Alyssa Y. Chen, MD, MPH**, Advanced Clinical: Medical Specialist

